# Re-weighted estimation of the transition probability density for second-order diffusion processes

**DOI:** 10.1371/journal.pone.0333958

**Published:** 2025-10-16

**Authors:** Yue Li, Yunyan Wang, Mingtian Tang

**Affiliations:** 1 School of Science, Jiangxi University of Science and Technology, Ganzhou, Jiangxi, People’s Republic of China; 2 Key Laboratory of Low Dimensional Quantum Materials and Sensor Devices of Jiangxi Education Institutes, Ganzhou, Jiangxi, People’s Republic of China; University of Southern California, UNITED STATES OF AMERICA

## Abstract

The transition probability density of second-order diffusion processes plays a fundamental role in statistical inference and practical applications such as financial derivatives pricing. This paper combines nonparametric Nadaraya-Watson kernel smoothing and local linear smoothing techniques to devise a re-weighted estimator for the transition probability density of second-order diffusion processes. The proposed estimator effectively addresses the persistent boundary bias inherent in Nadaraya-Watson estimation while preserving the nonnegativity constraint essential for probability densities. Under standard regularity conditions, we establish the asymptotic properties of the proposed estimator, demonstrating its theoretical superiority over existing approaches. Furthermore, Monte Carlo simulations show that the new estimator has better performance than Nadaraya-Watson estimator and local linear estimator.

## Introduction

Given the widespread recognition of diffusion models in the fields of finance and economics, diffusion processes are commonly employed to describe economic variables that evolve over time, such as stock prices, stock yields, and options futures [1,2]. The general diffusion process can be mathematically expressed through the following stochastic differential equation:

$$dX_{t}=\mu (X_{t})dt+\sigma (X_{t})dB_{t},$$ (1)

where $\left\{B_{t},t\geq 0\right\}$ represents a standard Brownian motion, μ(·) and σ(·) are the drift coefficient and diffusion coefficient of the process *X*_*t*_, respectively. Due to the fact that the diffusion process (1) is driven by a Brownian motion, its sample paths exhibit unbounded variation and are nowhere differentiable, thus posing challenges in modeling integrated and differentiated processes. As is well known, the integral stochastic process offers a superior explanation of modern econometric phenomena that manifest as the accumulation of past perturbations. For instance, in a discrete-time context, consider a unit root process $\left\{y_{t},t=0,1,2,\cdots \right\}$ where $y_{t}=\alpha +y_{t-1}+\varepsilon_{t}\;(\varepsilon_{t}\sim i.i.d.\;N(0,1))$. Notice that *y*_*t*_ can be written as


$$y_{t}=y_{0}+\sum_{k=1}^{t}x_{k},$$

where $x_{t}=\alpha +\varepsilon_{t}$. It is evident that the time series $\left\{y_{t},t=0,1,2,\cdots \right\}$ exhibits inherent nonstationarity. To overcome this limitation, a common strategy is to employ differential methods. For instance, for the unit root process {*y*_*t*_}, first-order differencing $\Delta y_{t}=y_{t}$ − *y*_*t*−1_ can transform the series into a stationary sequence, which is more convenient for further analysis and modeling.

For the continuous case, reference [3] developed the second-order diffusion process, which is defined by the following second-order stochastic differential equation based on [4,5]:

$$\left\{ \begin{array}{c} dY_{t}=X_{t}dt,\\ dX_{t}=\mu (X_{t})dt+\sigma (X_{t})dB_{t}, \end{array}\right.$$ (2)

where *X*_*t*_ is, by hypothesis, a stationary process, $\left\{B_{t},t\geq 0\right\}$ is a standard Brownian motion, μ(·) and σ(·) denote the drift coefficient and diffusion coefficient respectively. Furthermore, {*Y*_*t*_} is a differentiable process that can be expressed as $Y_{t}=Y_{0}$  +  $\int_{0}^{t}X_{s}ds.$ The process (2) is analogous to the unit root model and has been applied in finance and physics. Integrals of diffusion processes play a fundamental role in fields such as finance, engineering, and physics. For instance, in financial contexts, they can model asset returns tied to stock prices or exchange rates, capturing the stochastic evolution of market dynamics, as illustrated in works such as [3]. Additionally, in physical systems, *X*_*t*_ may represent the velocity of a particle and *Y*_*t*_ its corresponding position, while in paleoclimatology, such models help reconstruct paleotemperatures from ice-core data, as demonstrated in [6].

Second-order diffusion processes are fundamental in modeling complex stochastic dynamics across disciplines such as mathematical finance (e.g., capturing stochastic volatility in option pricing) and statistical physics (e.g., describing particle motion with memory effects). The transition probability density quantifies the likelihood of state transitions, and is a cornerstone for risk assessment, prediction, and model calibration.

However, if the unknown functions in the models cannot be accurately estimated, they cannot be further utilized, so the statistical inference of the model (2) is a prerequisite for its practical application. Compared with parametric methods, nonparametric estimation captures nonlinear relationships without pre-defined function forms, providing greater flexibility and robustness. Regarding the nonparametric estimation of the second-order diffusion process (2), it is worth noting that for a fixed sampling interval, we can observe $\left\{Y_{t_{i}},i=1,2,\cdots \right\}$, which is the cumulation effect of all past perturbations. However, the instantaneous value of *X* in model (2) at time *t*_*i*_ cannot be directly derived from these observations $Y_{t_{i}}$. In addition, the condition distribution of *Y* is usually unknown, even if the distribution of *X* is known, thereby nonparametric estimation based solely on {*Y*_*t*_} is not feasible. For i=1,2,⋯, let $t_{i}=i\Delta_{n}$ (where $\Delta_{n}=t_{i}$ − *t*_*i*−1_), as demonstrated by [3], with discrete-time observations $\left\{Y_{i\Delta_{n}},i=1,2,\cdots ,n\right\}$ and the relationship


$$Y_{i\Delta_{n}}-Y_{(i-1)\Delta_{n}}=\int_{0}^{i\Delta_{n}}X_{u}du-\int_{0}^{(i-1)\Delta_{n}}X_{u}du=\int_{(i-1)\Delta_{n}}^{i\Delta_{n}}X_{u}du,$$

we can approximate the value of *X* at $t_{i}=i\Delta_{n}$ using


$${\tilde{X}}_{i\Delta_{n}}=\frac{Y_{i\Delta_{n}}-Y_{(i-1)\Delta_{n}}}{\Delta_{n}},\;\;\Delta_{n}=t_{i}-t_{i-1}.$$

The accuracy of ${\tilde{X}}_{i\Delta_{n}}$ as a proxy for $X_{i\Delta_{n}}$ depends on the magnitude of $\Delta_{n}$. Throughout the paper, our estimation procedures depend on the sample $\left\{{\tilde{X}}_{i\Delta_{n}},i=1,2,\cdots ,n\right\}.$

For a review of nonparametric statistical inference of the second-order diffusion model (2), [3] developed the Nadaraya-Watson estimation of μ(·) and σ(·), and [7] proposed local linear estimators for the two infinitesimal coefficients. Combining the advantages of these two methods, [8] constructed the re-weighted estimator for the diffusion coefficient. [9] considered a generalized likelihood ratio test of the diffusion coefficient in the second-order diffusion model, and [10] proposed a new nonparametric estimator to correct the bias of the kernel estimator for diffusion coefficient in this model. Furthermore, [11] improved the conditions of [3] and established the strong consistency of the nonparametric kernel estimator.

Transition probability density function can fully characterize the dynamic process of the second-order diffusion model and help solve practical challenges such as financial asset pricing and portfolio selection. Consequently, the estimation of transition probability density has attracted widespread attention. [12] considered the kernel estimator of conditional density in nonparametric regression model, while [13] presented a local linear smoothing method that can be directly used to estimate the transition probability density of diffusion process. [14] adopted the conditional distribution smoother of [15], introduced a re-weighted Nadaraya-Watson smoother of the conditional density function. [16] proposed a new flexible nonparametric estimation technique for conditional density of time series models, which utilizes a regression-based approach and inherits the convergence rates of the selected regression methodology.

Beyond classical nonparametric approaches like Nadaraya-Watson and local linear estimation, modern methods for density estimation include adaptive bandwidth kernel estimation, log-spline density estimation, and wavelet density estimation. Adaptive bandwidth kernel estimation dynamically optimizes smoothing scales based on local data features [17], which can improve local estimation accuracy. However, in the second-order diffusion process, the random fluctuations of state variables (e.g., heteroscedasticity induced by the diffusion term σ(x)) can easily lead to excessive iteration of bandwidth selection, amplifying the negative effects of discrete observation errors, and making it difficult to balance the smoothing requirements in both conditioning variable *x* (current state) and response variable *y* (future state) directions. Log-spline density estimation achieves nonnegative density constraints through logarithmic transformation and spline basis function approximation, and demonstrates notable advantages in handling multimodal distributions and boundary issues [18]. Nevertheless, it requires preset fixed spline knots and cannot adaptively capture the locally time-varying structure of the transition probability density in second-order diffusion processes (e.g., abrupt changes in density morphology at the turning points of the drift term). Wavelet density estimation relies on multiscale decomposition to optimize the processing of abrupt data [19]. However, its theoretical convergence depends on the stationarity of data and the global completeness of orthogonal basis functions, making it difficult to adapt to the nonstationarity of second-order diffusion processes caused by the drift term μ(x) and diffusion term σ(x). Furthermore, it tends to introduce additional high-frequency noise in the approximation error of state variables (e.g., the deviation between the observed ${\tilde{X}}_{i\Delta_{n}}$ and the true $X_{i\Delta_{n}}$). The limitations of these methods in terms of scenario adaptability for transition probability density estimation of second-order diffusion processes highlight the necessity of developing targeted estimation methods.

In this paper, we develop a novel nonparametric estimation for the transition probability density function in second-order diffusion process. However, estimating the transition probability density of second-order diffusion processes is inherently challenging. Nonparametric estimation requires balancing bias and variance, and traditional methods have key limitations while laying the foundation. The Nadaraya-Watson estimator treats boundary points and interior points uniformly, which cannot explain the unique boundary conditions of the state space for the second-order diffusion process, resulting in boundary distortion. The fixed bandwidth limitation further limits its adaptability; A single bandwidth cannot capture the local and global features of transition probability density optimally, resulting in over smoothing (masking fine-grained transition patterns) or under smoothing (introducing too much noise). In addition, when the estimator strives to conform to the specific curvature and boundary behavior inherent in second-order diffusion process dynamics, significant asymptotic deviations may occur. The local linear estimation method solves some bias problems through local polynomial fitting, but it sacrifices a key characteristic of density estimation: nonnegativity. According to the definition, density estimation must be nonnegative, but local linear methods may generate false negative values in areas of low data density or complex boundary conditions in the pursuit of reducing bias, making them unsuitable for estimation of transition probability density in second-order diffusion processes.

To overcome these limitations, we turn to the re-weighted method, which has shown promise in other estimation contexts but has never been tailored to the unique requirements of transition probability density estimation for second-order diffusion processes. Our extension of the re-weighted method was initially proposed by [20] in a more general estimation framework. The re-weighted method we propose is not a mere transplantation of existing techniques but a specialized adaptation for transition probability density estimation of second-order diffusion processes. We recognize that the structure of second-order diffusion processes, with their specific stochastic differential equations governing state transitions, imposes different requirements on the estimator. The proposed estimator combines the bias advantage of local polynomial smoothing with the interpretability of classical kernel methods, while ensuring the nonnegativity of density estimation.

The re-weighted method has similarities with the Nadaraya-Watson smoothing method, but it introduces a new weighting scheme that subtly improves the Nadaraya-Watson estimation. [21] used it to investigate nonparametric estimation of regression functions, while [22] used it to estimate the volatility function of diffusion model. [23] developed a modified version of Nadaraya-Watson estimation for the infinitesimal conditional expectation of the second-order jump diffusion model, and [24] considered this re-weighted method for regression mean estimation. Furthermore, [25] utilized this method to estimate the conditional density in right-censored models.

The organization of this paper is outlined as follows. The Methods section establishes the re-weighted estimator for the transition probability density of the second-order diffusion model. The Asymptotic results section lists the necessary conditions and presents asymptotic results of the proposed estimator. The simulation experiment is presented in the Simulation section. The detailed proofs of the main results are given in the Auxiliary results and proofs section. The last section is a conclusion.

## Methods

Throughout this paper, we assume that *X* on a space D=(l,r), −∞≤l<r≤+∞. The unknown function p(y|x) is the transition density of $X_{t_{i+1}}=y$ given $X_{t_{i}}=x$ in the model (2), it can be estimated based on joint density *p*(*x*, *y*) and marginal density *p*(*x*) (where p(·) is assumed positive at *x*) since we have


$$p(y|x)\text{ = }\frac{p(x,y)}{p(x)}.$$

The kernel function K(·) is a nonnegative and symmetric density function on ℝ, satisfies


$$\int_{\mathbb{R}}K(u)du=1,\int_{\mathbb{R}}uK(u)du=0,\int_{\mathbb{R}}u^{2}K(u)du<\infty .$$

Let *h*_*n*_ be bandwidth, and $K_{h_{n}}(\cdot )=K({\cdot /h}_{n})/h_{n}$. As $h_{n}\to 0$, we have

$$E\left[K_{h_{n}}(y-{\tilde{X}}_{(i+1)\Delta_{n}})|X_{i\Delta_{n}}=x\right]=p(y|x)+O(h_{n}^{2}).$$ (3)

In fact, by Taylor’s expansion, we have


$$E\left[K_{h_{n}}(y-X_{(i+1)\Delta_{n}})|X_{i\Delta_{n}}=x\right]$$

$$=\int_{\mathbb{R}}K_{h_{n}}(y-u)p(u|x)du$$

$$=\int_{\mathbb{R}}K_{h_{n}}(y-u)\left[p(y|x)+p^{\prime }(y|x)(u-y)+\frac{p^{\prime \prime }(y|x)}{2}{\left(u-y\right)}^{2}\right]du$$

$$=p(y|x)\int_{\mathbb{R}}K(v)dv+p^{\prime }(y|x)\int_{\mathbb{R}}vK(v)dv+\frac{p^{\prime \prime }(y|x)h_{n}^{2}}{2}\int_{\mathbb{R}}v^{2}K(v)dv$$

$$=p(y|x)+O(h_{n}^{2}).$$

Furthermore, by Taylor’s expansion and Hölder’s inequality, we have


$$\begin{array}{c} \;\;\;E\left[K_{h_{n}}(y-{\tilde{X}}_{(i+1)\Delta_{n}})-K_{h_{n}}(y-X_{(i+1)\Delta_{n}})\right]\\ =\frac{1}{h_{n}}E\left[K^{\prime }(\zeta_{ni})\frac{{\tilde{X}}_{(i+1)\Delta_{n}}-X_{(i+1)\Delta_{n}}}{h_{n}}\right]\\ \leq \left(\frac{1}{h_{n}}E{\left[{\left|K^{\prime }(\zeta_{ni})\right|}^{\alpha }\right]}^{1/\alpha }\right)\times \left(\frac{1}{h_{n}}E{\left[{\left|{\tilde{X}}_{(i+1)\Delta_{n}}-X_{(i+1)\Delta_{n}}\right|}^{\beta }\right]}^{1/\beta }\right)\times E{\left[{\left|1\right|}^{\gamma }\right]}^{1/\gamma }, \end{array}$$

where $\zeta_{ni}=\theta (\left(y-X_{(i+1)\Delta_{n}}\right)/h_{n})+(1-\theta )(\left(y-{\tilde{X}}_{(i+1)\Delta_{n}}\right)/h_{n}),0\leq \theta \leq 1$, and 1 / *α* + 1 / *β* + 1 / *γ* = 1. Selecting α=4,β=4,γ=2, and assuming that


$$\frac{1}{h_{n}}E{\left[{\left|K^{\prime }(\zeta_{ni})\right|}^{4}\right]}^{1/4}<\infty ,$$

by Lemma 1 in the Auxiliary results and proofs section we have


$$\frac{1}{h_{n}}E{\left[{\left|{\tilde{X}}_{(i+1)\Delta_{n}}-X_{(i+1)\Delta_{n}}\right|}^{4}\right]}^{1/4}\to 0,$$

so $E\left[K_{h_{n}}(y-{\tilde{X}}_{(i+1)\Delta_{n}})-K_{h_{n}}(y-X_{(i+1)\Delta_{n}})\right]\to 0.$

Let $\alpha_{j}={\left(p(y|x)\right)}^{\left(j\right)}/j!,j=0,1,2,\cdots ,p,$ neglecting the higher order term in (3), which suggests that p(y|x) can be regarded as a regression of $K_{h_{n}}(y$ − ${\tilde{X}}_{(i+1)\Delta_{n}})$ on $X_{i\Delta_{n}}$, so by selecting $\alpha_{j}$ minimize the weighted sum of squares


$$\sum_{i=1}^{n}{\left(K_{h_{n}}(y-{\tilde{X}}_{(i+1)\Delta_{n}})-\sum_{j=0}^{p}\alpha_{j}{\left({\tilde{X}}_{i\Delta_{n}}-x\right)}^{j}\right)}^{2}K_{h_{n}}(x-{\tilde{X}}_{i\Delta_{n}}),$$

we can get the local polynomial estimator of the transition probability density for model (2).

When *p* = 0, the estimator is known as the Nadaraya-Watson (NW) kernel estimator:


$${\hat{p}}_{1}(y|x)=\frac{\sum_{i=1}^{n}K_{h_{n}}(x-{\tilde{X}}_{i\Delta_{n}})K_{h_{n}}(y-{\tilde{X}}_{(i+1)\Delta_{n}})}{\sum_{i=1}^{n}K_{h_{n}}(x-{\tilde{X}}_{i\Delta_{n}})}.$$

When *p* = 1, we can get local linear (LL) estimator as follows:


$${\hat{p}}_{2}(y|x)=\sum_{i=1}^{n}\omega_{i}^{LL}(x)K_{h_{n}}(y-{\tilde{X}}_{(i+1)\Delta_{n}}),$$

where


$$\omega_{i}^{LL}(x)=\frac{K_{h_{n}}(x-{\tilde{X}}_{i\Delta_{n}})\left\{T_{n,2}-(x-{\tilde{X}}_{i\Delta_{n}})T_{n,1}\right\}}{\left(T_{n,0}T_{n,2}-T_{n,1}^{2}\right)},$$

$$T_{n,j}=\sum_{i=1}^{n}K_{h_{n}}(x-{\tilde{X}}_{i\Delta_{n}})(x-{\tilde{X}}_{i\Delta_{n}}{)}^{j},j=0,1,2.$$

According to minimum mean square theory, $\left\{\omega_{i}^{LL}(x),i=1,2,\cdots ,n\right\}$ satisfies


$$\sum_{i=1}^{n}{\omega_{i}}^{LL}(x)=1,\;\;\sum_{i=1}^{n}\left(x-{\tilde{X}}_{i\Delta_{n}}\right)\omega_{i}^{LL}(x)=0.$$

Reference [17] indicates that these moment conditions make the local linear estimator superior to the Nadaraya-Watson estimator in terms of boundary deviation. In order to combine the advantages of Nadaraya-Watson and local linear methods, this paper considers re-weighted methods for transition probability density. This involves making minimal adjustments to the weights of Nadaraya-Watson estimator to ensure the nonnegativity while adhering to the conditions of $\omega_{i}^{LL}(x)$. The specific form of the re-weighted estimator is detailed as follows:


$$\hat{p}(y|x)=\frac{\sum_{i=1}^{n}{\omega_{i}}^{RNW}(x)K_{h_{n}}(x-{\tilde{X}}_{i\Delta_{n}})K_{h_{n}}(y-{\tilde{X}}_{(i+1)\Delta_{n}})}{\sum_{i=1}^{n}{\omega_{i}}^{RNW}(x)K_{h_{n}}(x-{\tilde{X}}_{i\Delta_{n}})},$$

where the weight function $\left\{{\omega_{i}}^{RNW}(x),i=1,2,\ldots ,n\right\}$ satisfies


$$\underset{\left\{\omega_{i}\right\}}{\max}\frac{1}{n}\sum_{i=1}^{n}\log(n\omega_{i})$$

subject to


$$\sum_{i=1}^{n}\omega_{i}=1,\omega_{i}\geq 0,$$

$$\sum_{i=1}^{n}\omega_{i}\cdot (x-{\tilde{X}}_{\left(i+1\right)\Delta_{n}})K_{h_{n}}(x-{\tilde{X}}_{i\Delta_{n}})=0.$$

By using Lagrange multipliers method, we can obtain


$${\omega_{i}}^{RNW}(x)=\frac{1}{n(1+\lambda (x-{\tilde{X}}_{\left(i+1\right)\Delta_{n}})(K_{h_{n}}(x-{\tilde{X}}_{i\Delta_{n}})))},$$

where *λ* satisfies


$$\frac{1}{n}\sum_{i=1}^{n}\frac{\left(x-{\tilde{X}}_{\left(i+1\right)\Delta_{n}})K_{h_{n}}(x-{\tilde{X}}_{i\Delta_{n}}\right)}{1+\lambda (x-{\tilde{X}}_{\left(i+1\right)\Delta_{n}})K_{h_{n}}(x-{\tilde{X}}_{i\Delta_{n}})}=0.$$

For more details, readers can refer to [8, pp. 1132–1134].

In fact, The kernel function $K_{h_{n}}(x$ − ${\tilde{X}}_{i\Delta_{n}})$ acts as a smoothing filter that assigns higher weights to historical states ${\tilde{X}}_{i\Delta_{n}}$ which are close to the current state *x*. Correspondingly, $K_{h_{n}}(y$ − ${\tilde{X}}_{(i+1)\Delta_{n}})$ weights the future states *y* that are proximate to the observed subsequent state ${\tilde{X}}_{(i+1)\Delta_{n}}$. The re-weighted mechanism $\omega_{i}^{RNW}(x)$ further adjusts these kernel weights by incorporating a moment constraint, e.g.,


$$\sum_{i=1}^{n}\omega_{i}\cdot (x-{\tilde{X}}_{\left(i+1\right)\Delta_{n}})K_{h_{n}}(x-{\tilde{X}}_{i\Delta_{n}})=0,$$

which effectively corrects for boundary bias. This adjustment proves particularly critical when handling edge cases, such as abrupt yet physically plausible state transitions (e.g., rapid temperature fluctuations), as it prioritizes data sequences that are both statistically influential and dynamically consistent with real-world constraints.

In essence, the re-weighted estimator integrates local data proximity (enforced by the kernels) with structural regularity (enforced by re-weighted method) to produce a physically realistic and statistically efficient estimate of the transition probability from state *x* to *y*.

## Asymptotic results

The main results of this paper are based on the following conditions.

A1. (i) Let the state space of random process *X* be D=(l,r). Let *z*_0_ be any point in interval D, and $s(z)=\exp\left\{-\int_{z_{0}}^{z}\frac{2\mu (x)}{\sigma^{2}(x)}dx\right\}$ be the scale density function. In addition, for x∈D, $l\;<\;x_{1}\;<\;x_{2}<r$, we assume that


$$S(l,x]=\underset{x_{1}\to l}{\lim}\int_{x_{1}}^{x}s(u)du=\infty ,\;\;\;S[x,r)=\underset{x_{2}\to r}{\lim}\int_{x}^{x_{2}}s(u)du=\infty ;$$

(ii) For x∈D, $\int_{l}^{r}m(x)dx<\infty$, where $m(x)=(\sigma^{2}(x)s(x){)}^{-1}$ is the speed density function;

(iii) *X*_0_ = *x* has distribution *P*^0^, where *P*^0^ is the invariant distribution of process *X*.

Remark 1. Assumption A1 assures that *X* is a stationary process ([3]).

A2. Suppose that


$$\underset{x\to r}{\lim\sup}\left(\frac{\mu (x)}{\sigma (x)}-\frac{\sigma^{\prime }(x)}{2}\right)<0,$$

$$\underset{x\to l}{\lim\sup}\left(\frac{\mu (x)}{\sigma (x)}-\frac{\sigma^{\prime }(x)}{2}\right)>0.$$

Remark 2. Assumption A2 assures that *X* is *ρ*-mixing and *α*-mixing, and under assumptions A1-A2, we can obtain that $\left\{{\tilde{X}}_{i\Delta },i=0,1,\cdots \right\}$ is a stationary process, readers can refer to [3] for details.

A3. (i) The marginal density function *p*(*x*) > 0 is a bounded continuous function and has a continuous first-order derivative;

(ii) The transition probability density function p(y|x) is a bounded continuous function with continuous second-order partial derivatives.

(iii) The joint density *p*(*x*,*y*) is bounded by an independent constant.

A4. Kernel function *K*(*x*) is a continuously differentiable, symmetric density function with bounded support and satisfies


$$\int_{\mathbb{R}}K(u)du=1,\;\int_{\mathbb{R}}uK(u)du=0,\;\;\int_{\mathbb{R}}u^{2}K(u)du<\infty ,\int u^{2}K^{2}(u)du<\infty ,\;\;\;\left|u\right|K(u)\to 0\;(\left|u\right|\to \infty ),\int_{\mathbb{R}}{\left|K^{\prime }(u)\right|}^{2}du<\infty ,\;\;\;\int_{\mathbb{R}}K^{2}(u)du<\infty .$$

Remark 3. The selection of kernel function is a fundamental consideration in nonparametric estimation, and its impact on the proposed re-weighted estimation deserves clear discussion. In practice, various types of kernels can be used, and any density function can serve as a valid kernel, even nonpositive functions have been proven theoretically feasible (see [26]). For the purpose of this study, we restrict our attention to classical positive symmetric kernels (e.g., the Gaussian kernel used in our simulation), due to their computational simplicity and widespread adoption in the literature. It is worth noting that both theoretical analysis and empirical evidence indicate that specific kernel choices have only a weak impact on the performance of kernel based estimators. This insensitivity is due to the asymptotic properties of the estimator, including bias and variance, as described in Theorem 1, which mainly depend on the bandwidth *h*_*n*_ and kernel moments, rather than the exact functional form of the kernel.

Practically, the Gaussian kernel is more popular due to its smoothness and ease of implementation, especially in dealing with boundary effects. For applications that require computational efficiency, alternative kernels (e.g., Epanechnikov) may be substituted without altering the asymptotic properties, as long as the regularity conditions in Assumption A4 are satisfied.

A5. $\underset{h_{n}\to 0}{\lim}\frac{1}{h_{n}^{m}}E\left({\left|mK^{\left(m-1\right)}(\xi_{n,i})\right|}^{4}\right)<\infty$, where *m* is a positive integer, $\xi_{ni}=\theta \left(\frac{x-X_{i\Delta_{n}}}{h_{n}}\right)$ $+(1-\theta )\left(\frac{x-{\tilde{X}}_{i\Delta_{n}}}{h_{n}}\right)$, 0≤θ≤1.

A6. (i) μ(x) and σ(x) have continuous derivatives of order 4 and satisfy $\left|\mu (x)\right|\leq C(1+\left|x\right|{)}^{\tau }$ and $\left|\sigma (x)\right|\leq C(1+\left|x\right|{)}^{\tau }$ for some τ>0.

(ii) $E\left[X_{0}^{r}\right]<\infty$, where r=max{4τ,1+3τ,−1+5τ,−2+6τ}.

Remark 4. Assumption A6 imposes moment bounds on the drift coefficient μ(x), diffusion coefficient σ(x) and the initial state *X*_0_, which are standard in the literature on nonparametric estimation for diffusion processes (e.g., [3,8]). This assumption is related to the application of Lemma 1 (Auxiliary results and proofs section).

A7. Let ${ℱ}_{i}^{j}$ denote the *σ*-field generated by $\left\{X_{t}:i\leq t\leq j\right\}$, for all s,t≥1, the mixing coefficient $\alpha (t)=\sup\{\left|P(A\cap B-P(A)P(B)\right|:A\in {ℱ}_{1}^{s},B\in {ℱ}_{s+t}^{\infty }\}$ satisfies the following conditions:

(i) For some constant *δ*, 0<δ<1, and a>δ, $\sum_{i=1}^{\infty }i^{a}\alpha^{\delta }\left(i\right)<\infty$;

(ii) Assume that there exists a sequence of positive integers *s*_*n*_ such that $s_{n}\to \infty$, $s_{n}=o(\sqrt{nh_{n}^{2}})$, $(n/h_{n}^{2}{)}^{1/2}\alpha \left(s_{n}\right)\to 0$.

Remark 5. Assumption A7 imposes *α*-mixing condition on {*X*_*t*_}, a large class of strongly mixing random variables with mixing coefficient α(t) satisfies these conditions are included.

A8. (i) $\Delta_{n}\to 0$, $h_{n}\to 0$, $nh_{n}^{2}\to \infty$, Δn/Δnhnhn→0 as n→∞;

(ii) Assume that n→∞, $\Delta_{n}\to 0$ and $h_{n}\to 0$ such that Δn/Δn(nhn4)(nhn4)→0, and


(nΔn/nΔnhn)(Δnlog(1/1ΔnΔnhn)(Δnlog(1/1ΔnΔn))1/122→0.

Remark 6. (i) The term $\sqrt{\Delta_{n}\log(1/\Delta_{n})}$ originates from the modulus continuity of the Brownian motion paths (see [27]), which characterizes the asymptotic magnitude of Brownian motion oscillations at microscopic time scales. It is prevalent in many other studies, serving as a tool to control discretization errors, and it ensures the asymptotic properties of the estimator remain valid as $\Delta_{n}\to 0$, a result well-established in the literature (see [3, Theorem 3]).

(ii) The bandwidth *h*_*n*_ is a key hyperparameter in nonparametric kernel estimation, it controls the smoothness of the final curve estimation, and its selection directly affects the bias variance trade-off of the proposed re-weighted estimator. The selection of bandwidth requires balancing two conflicting objectives: a smaller bandwidth can reduce bias by closely adapting to local data structures, but it will increase variance due to limited sample information, while a larger bandwidth can smooth noise but may over average the fine-scale features of the data, thereby introducing bias.

Practically, cross-validation (CV) or plug-in methods can be employed to optimize bandwidth selection. As to how to choose the bandwidth the book [17] is recommended.

Theorem 1. Under A1-A8, let $\sigma_{K}^{2}$ $=\int u^{2}K(u)du$, $R\left(K\right)=\int K^{2}(u)du$, for ∀x,y∈D, as n → ∞, we have

(i) $\hat{p}(y|x)-p(y|x)=\frac{1}{2}\sigma_{K}^{2}h_{n}^{2}\left(\frac{\partial^{2}p(y|x)}{\partial x^{2}}+\frac{\partial^{2}p(y|x)}{\partial y^{2}}\right)+O_{p}((nh_{n}^{2}{)}^{-\frac{1}{2}}).$

(ii) Furthermore, if $nh_{n}^{6}\to c(c\neq 0)$ and $nh_{n}^{2}\Delta_{n}^{2}\to 0$, then


$$\sqrt{nh_{n}^{2}}\left(\hat{p}(y|x)-p(y|x)-\frac{1}{2}\sigma_{K}^{2}h_{n}^{2}\left(\frac{\partial^{2}p(y|x)}{\partial x^{2}}+\frac{\partial^{2}p(y|x)}{\partial y^{2}}\right)\right)\overset{D}{\to }N\left(0,\frac{R^{2}(K)p(y|x)}{p(x)}\right),$$

where $\overset{D}{\to }$ means convergence by distribution.

Remark 7. (i) Under Theorem 1, we know that the expectation and variance of the estimator $\hat{p}(y|x)$ are as follows:


$$E\left[\hat{p}(y|x)-p(y|x)\right]=\frac{1}{2}\sigma_{K}^{2}h_{n}^{2}\left(\frac{\partial^{2}p(y|x)}{\partial x^{2}}+\frac{\partial^{2}p(y|x)}{\partial y^{2}}\right)+o_{p}(h_{n}^{2}),$$

$$V\text{ar}(\hat{p}(y|x))=(nh_{n}^{2}{)}^{-1}\frac{R^{2}(K)p(y|x)}{p(x)}+o_{p}((nh_{n}^{2}{)}^{-1}).$$

Compared to [28, Theorem 1, p. 5], the re-weighted estimator $\hat{p}(y|x)$ shares the variance of the Nadaraya-Watson kernel estimator ${\hat{p}}_{1}(y|x)$, the difference in the asymptotic mean squared error depends on the magnitude of the deviation between them. According to [28, p. 5], under the conditions A1-A6 and A8, for ∀x,y∈D, the approximate asymptotic bias of the Nadaraya-Watson estimator is as follows:


$$Bias({\hat{p}}_{1}(y|x))=\frac{1}{2}\sigma_{K}^{2}h_{n}^{2}\left(\frac{\partial^{2}p(y|x)}{\partial x^{2}}+2\frac{p^{\prime }(x)}{p(x)}\frac{\partial p(y|x)}{\partial x}+\frac{\partial^{2}p(y|x)}{\partial y^{2}}\right).$$

Obviously, the re-weighted estimator exhibits superior bias properties in comparison to the Nadaraya-Watson estimator. Firstly, ${\hat{p}}_{1}(y|x)$ incorporates an additional deviation item, $2\frac{p^{\prime }(x)}{p(x)}\frac{\partial p(y|x)}{\partial x}$ in contrast to $\hat{p}(y|x)$. When either $\left|\frac{p^{\prime }(x)}{p(x)}\right|$ or $\left|\frac{\partial p(y|x)}{\partial x}\right|$ assumes a large value, the deviation of ${\hat{p}}_{1}(y|x)$ tends to be larger. Consequently, under identical conditions, the asymptotic bias of the re-weighted estimator is smaller than that of the Nadaraya-Watson estimator. Nadaraya-Watson estimator ${\hat{p}}_{1}(y|x)$ exhibits systematic estimation bias in the boundary region of the data distribution, known as boundary effects, which result in significant deviations between the estimated results and the true values. In a sense, $\hat{p}(y|x)$ can be regarded as the result of bias reduction of ${\hat{p}}_{1}(y|x)$. For a detailed theoretical derivation and in-depth analysis of the bias term, readers can refer to [17], which systematically explains the formation and properties of the bias term in nonparametric estimation.

(ii) Theorem 1 also suggests the criterion for selecting the smoothing bandwidth:


$$h_{n}=Cn^{-1/6},$$

which minimizes the asymptotic mean squared error of the re-weighted estimator. Here *C* is a constant and can be determined via the cross-validation method. It is noteworthy that, in contrast to the conventional *n*^−1/5^ rate prevalent in univariate density estimation, the optimal bandwidth rate here is *n*^−1/6^, as smoothing is required in both *x* and *y* directions.

## Simulation

In this section, we perform Monte Carlo experiments to evaluate the estimation performance of the re-weighted estimator. The experiment is based on the following second-order stochastic differential equation:


$$\left\{ \begin{array}{c} dY_{t}=X_{t}dt\\ dX_{t}=-10X_{t}dt+\sqrt{0.1+0.1{X_{t}}^{2}}dB_{t}. \end{array}\right.$$

In the simulation, we select a fixed observation time interval, i.e., t∈[0,T]=[0,10], which is subsequently partitioned into 5,000 equal segments, resulting in a corresponding sampling frequency of $\Delta =\Delta_{n}=\frac{T}{n}=0.002$. To approximate the values of *X*_*t*_ within this interval, we use the Euler-Maruyama method, a widely recognized numerical technique for approximating solutions of stochastic differential equations:


$$X_{t+1}=X_{t}-10X_{t}\Delta +\sqrt{0.1+0.1{X_{t}}^{2}}\left(\sqrt{\Delta }N\left(0,1\right)\right).$$

In this section, we consider Gaussian kernel K(u)=exp(−u2/−u22)2)/exp(−u2/−u22)2)2π2π and common bandwidth $h_{n}=1.06Sn^{-1/6}$, where *S* is the standard deviation of the sample $\left\{{\tilde{X}}_{i\Delta_{n}},i=1,2,\cdots ,n\right\}$.

**Fig 1:**
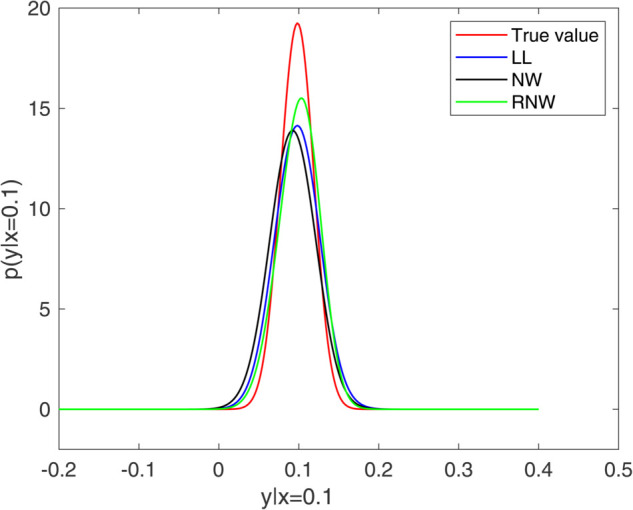
Transition probability density estimators at x=0.1. (Nadaraya-Watson estimator (NW), local linear estimator (LL) and re-weighted estimator (RNW)).

**Fig 2:**
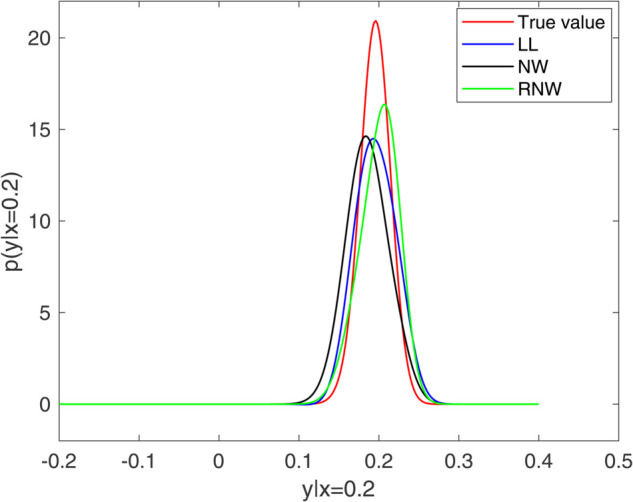
Transition probability density estimators at x=0.2. (Nadaraya-Watson estimator (NW), local linear estimator (LL) and re-weighted estimator (RNW)).

Figs 1 and 2 present a comparative analysis of estimation performance at *x* = 0.1 and x = 0.2, where the exact values are obtained by setting the sampling frequency Δ=0.0005. As shown in the figures, the re-weighted estimator exhibits a similar symmetry to the true density and other estimators, and indicating a reduced bias compared to the Nadaraya-Watson estimator. This reduction of bias is attributed to the re-weighted estimator’s incorporation of the automatic bias correction mechanism of local linear estimation. Furthermore, the transition probability density attains its maximum value in proximity to the given value of *x*, and its skewness varies according to *x*, thus demonstrating the effectiveness of our proposed estimator.

To compare the performance of re-weighted estimator and local linear estimator in preserving the essential nonnegativity property of density estimates, we simulated the values of different transition probability density estimators at fixed *x* = 0.2. The results are presented in Table 1, which indicates that the local linear estimator exhibits negative results at certain design points, while the re-weighted estimator consistently provides the nonnegative values for transition probability density.

**Table 1:** The estimates of the transition probability density at some design points. (Nadaraya-Watson estimator ${\hat{p}}_{1}(y|x)$, local linear estimator ${\hat{p}}_{2}(y|x)$ and re-weighted estimator $\hat{p}(y|x)$)

*y*	p(y|x)	${\hat{p}}_{1}(y|x)$	${\hat{p}}_{2}(y|x)$	$\hat{p}(y|x)$
0	1.29E-15	3.47E-09	–2.17E-09	9.69E-12
0.04	1.90E-09	1.98E-05	–3.80E-06	4.43E-07
0.08	7.48E-05	0.0337	1.54E-02	0.0015
0.12	0.1261	2.1583	1.2456	0.2821
0.16	6.7173	7.5772	5.5410	4.1535
0.20	17.6938	12.8575	15.1596	16.6301
0.24	0.7011	2.4993	3.1591	3.9761
0.28	0.0002	0.0058	0.0076	0.0094
0.32	6.49E-11	1.09E-07	1.45E-07	1.75E-07
0.36	1.88E-20	1.44E-14	1.93E-14	2.31E-14
0.40	3.79E-33	1.28E-23	1.72E-23	2.05E-23

Table 2 compares the mean squared error (MSE) performance of the Nadaraya-Watson estimator (${\hat{p}}_{1}(y|x)$), local linear estimator (${\hat{p}}_{2}(y|x)$) and re-weighted estimator ($\hat{p}(y|x)$) of the transition probability density, indicating superior accuracy of the re-weighted estimator, while all estimators show improved performance with increasing sampling frequency.

**Table 2:** The MSE of transition probability density estimators. (Nadaraya-Watson estimator ${\hat{p}}_{1}(y|x)$, local linear estimator ${\hat{p}}_{2}(y|x)$ and re-weighted estimator ${\hat{p}}_{2}(y|x)$)

*y*	Δ=0.002			Δ=0.001		
	${\hat{p}}_{1}(y|x=0.1)$	${\hat{p}}_{2}(y|x=0.1)$	$\hat{p}(y|x=0.1)$	${\hat{p}}_{1}(y|x=0.1)$	${\hat{p}}_{2}(y|x=0.1)$	$\hat{p}(y|x=0.1)$
0	0.0101	0.0018	6.65E-04	2.59E-04	4.04E-05	3.16E-05
0.04	4.5094	1.9673	0.8577	0.8020	0.3179	0.2046
0.08	4.1702	1.1012	0.5647	1.7552	0.3545	0.1243
0.12	2.1562	2.0380	0.4618	1.1761	0.3763	0.1362
0.16	0.5386	1.0655	0.5275	0.1124	0.2237	0.1084
0.20	4.58E-04	0.0012	8.60E-05	1.37E-05	3.81E-05	3.31E-06
0.24	5.71E-08	1.50E-07	3.49E-09	1.32E-10	4.23E-10	1.36E-11
0.28	1.32E-12	3.20E-12	2.23E-14	2.65E-16	9.38E-16	5.87E-18
0.32	2.44E-18	5.95E-18	5.97E-21	1.97E-23	7.71E-23	6.40E-26
0.36	2.69E-25	7.34E-25	7.53E-29	1.44E-32	6.77E-32	1.49E-35
0.40	5.56E-34	2.61E-33	6.46E-38	5.13E-43	2.98E-42	9.40E-47

## Auxiliary results and proofs

Lemma 1 [3]. Let *Z* be a *d*-dimensional diffusion process determined by the following stochastic integral equation:


$$Z_{t}=Z_{0}+\int_{0}^{t}a(Z_{s})ds+\int_{0}^{t}b(Z_{s})dW_{s},$$

where $a(z)=[a_{i}(z){]}_{d\times 1}$ is a *d* × 1 vector, $[b_{ij}(z){]}_{d\times d}$ is a *d* × *d* diagonal matrix, and *W*_*t*_ is a *d* × 1 vector of independent Brownian motions. Suppose a(·) and b(·) have continuous partial derivatives of order 2*s*, *f*(*z*) is a continuous function with continuous partial derivative of order 2*s* + 2 defined on ${\mathbb{R}}^{d}$ and with values in ${\mathbb{R}}^{d}$, then


$$E[f(Z_{i\Delta })|Z_{(i-1)\Delta }]=\sum_{k=0}^{s}L^{k}f(Z_{(i-1)\Delta })\frac{\Delta^{k}}{k!}+R,$$

where *L* is a second-order differential operator determined by equation


$$L=\sum_{i=1}^{d}\mu_{i}(z)\frac{\partial }{\partial z_{i}}+\frac{1}{2}(\sigma_{11}^{2}(z)\frac{\partial^{2}}{\partial z_{1}^{2}}+\sigma_{22}^{2}(z)\frac{\partial^{2}}{\partial z_{2}^{2}}+...+\sigma_{dd}^{2}(z)\frac{\partial^{2}}{\partial z_{d}^{2}}),$$

and *R* is a random function of order $\Delta^{s+1}$, and


$$R=\int_{(i-1)\Delta }^{i\Delta }\int_{(i-1)\Delta }^{u_{1}}\int_{(i-1)\Delta }^{u_{2}}\cdots \int_{(i-1)\Delta }^{u_{s}}E[L^{s+1}f(Z_{u_{s+1}})|Z_{(i-1)\Delta }]du_{s+1}du_{s}\cdots du_{1}.$$

Remark 8. Here, we consider *d* = 2. For model (1.2), we have


$$L=x\frac{\partial }{\partial y}+\mu (x)\frac{\partial }{\partial x}+\frac{1}{2}\sigma^{2}(x)\frac{\partial^{2}}{\partial x^{2}}.$$

Based on Lemma 1, we can calculate a variety of mathematical expectations involving ${\tilde{X}}_{i\Delta_{n}}$, such as (12) and (13) (see the Auxiliary results and proofs section for details).

Lemma 2 [8]. Consider


$$\varepsilon_{1,n}=\frac{1}{n}\sum_{i=1}^{n}(K_{h_{n}}(x-{\tilde{X}}_{i\Delta_{n}}){)}^{m}g({\tilde{X}}_{i\Delta_{n}},{\tilde{X}}_{(i+1)\Delta_{n}}),$$

$$\varepsilon_{2,n}=\frac{1}{n}\sum_{i=1}^{n}(K_{h_{n}}(x-X_{i\Delta_{n}}){)}^{m}g({\tilde{X}}_{i\Delta_{n}},{\tilde{X}}_{(i+1)\Delta_{n}}).$$

where *g* is a measurable function defined on ℝ × ℝ, *m* is a positive integer. Assume that Δn/Δnhnhn→0 and A1, A4, A5 hold. If for any n∈N,i=1,2,..., $E\left[{\left(g({\tilde{X}}_{i\Delta_{n}},{\tilde{X}}_{(i+1)\Delta_{n}})\right)}^{2}\right]<\infty$, then $\left|\varepsilon_{1,n}-\varepsilon_{2,n}\right|\overset{P\;}{\to }0.$

Lemma 3 [8]. Let p(·) be the density function of the process *X*_*t*_, $\sigma_{K}^{2}=\int u^{2}K(u)du$, $v_{2}=\int u^{2}K^{2}(u)du$, under the conditions A1-A2, A4-A6, A8, we have


$$\lambda =\frac{h\sigma_{K}^{2}p^{\prime }(x)}{v_{2}p(x)}+O_{p}(h^{3}),\left|\lambda \right|=O_{p}(h).$$

Lemma 4 [14]. Consider the model (1), suppose that A1-A4, A6-A8 hold and $nh_{n}^{6}\to c(c\;\neq \;0)$, $\sigma_{K}^{2}=\int u^{2}K(u)du$, $R\left(K\right)=\int K^{2}(u)du$. Then, for ∀x,y∈D, n→∞, we have


$$\text{(i)}\;{\hat{p}}_{0}(y|x)-p(y|x)=\frac{1}{2}\sigma_{K}^{2}h_{n}^{2}\left(\frac{\partial^{2}p(y|x)}{\partial x^{2}}+\frac{\partial^{2}p(y|x)}{\partial y^{2}}\right)+O_{p}((\sqrt{nh_{n}^{2}}{)}^{-1}),$$

$$\text{(ii)}\;\sqrt{nh_{n}^{2}}\left({\hat{p}}_{0}(y|x)-p(y|x)-\frac{1}{2}\sigma_{K}^{2}h_{n}^{2}\left(\frac{\partial^{2}p(y|x)}{\partial x^{2}}+\frac{\partial^{2}p(y|x)}{\partial y^{2}}\right)\right)\overset{D}{\to }N\left(0,\frac{R^{2}(K)p(y|x)}{p(x)}\right),$$

where


$${\hat{p}}_{0}(y|x)=\frac{A_{0}(x,y)}{B_{0}(x)}=\frac{\sum_{i=1}^{n}{\omega_{i}}^{0}(x)K_{h_{n}}(x-X_{i\Delta_{n}})K_{h_{n}}(y-X_{(i+1)\Delta_{n}})}{\sum_{i=1}^{n}{\omega_{i}}^{0}(x)K_{h_{n}}(x-X_{i\Delta_{n}})},$$

with


$${\omega_{i}}^{0}(x)=\frac{1}{n(1+\lambda_{0}(x-X_{\left(i+1\right)\Delta_{n}})K_{h_{n}}(x-X_{i\Delta_{n}}))},$$

and $\lambda_{0}$ satisfies


$$\frac{1}{n}\sum_{i=1}^{n}\frac{\left(x-X_{\left(i+1\right)\Delta_{n}})K_{h_{n}}(x-X_{i\Delta_{n}}\right)}{1+\lambda_{0}(x-X_{\left(i+1\right)\Delta_{n}})K_{h_{n}}(x-X_{i\Delta_{n}})}=0,\;\;\left|\lambda_{0}\right|=O_{p}(h_{n}).$$

Remark 9. Lemma 4 restates Theorem 1 of [14] in the specific context of second-order diffusion processes, establishing the asymptotic properties of the idealized estimator ${\hat{p}}_{0}(y|x)$ based on the true (unobservable) process {*X*_*t*_}. This adaptation is rigorously validated under conditions with equivalent effects (mixing conditions A2 and A7, moment bounds A6, kernel smoothing conditions A4) and the infinitesimal moment of the process {*X*_*t*_}:


$$E\left[K_{h_{n}}(y-X_{(i+1)\Delta_{n}})|X_{i\Delta_{n}}=x\right]=p(y|x)+O(h_{n}^{2}).$$

A key distinction is that $X_{i\Delta_{n}}$ is unobservable in the second-order diffusion processes (2), and the proposed re-weighted estimator is based on ${\tilde{X}}_{i\Delta_{n}}=\frac{Y_{i\Delta_{n}}-Y_{(i-1)\Delta_{n}}}{\Delta_{n}}$, by using Lemma 4 and the difference of ${\tilde{X}}_{i\Delta_{n}}$ and $X_{i\Delta_{n}}$, the proof of Theorem 1 only requires proving the equivalence of the estimators between $\hat{p}(y|x)$ and ${\hat{p}}_{0}(y|x)$.

Proof of Theorem 1. (i) Let


$$A(x,y)=\sum_{i=1}^{n}{\omega_{i}}^{RNW}(x)K_{h_{n}}(x-{\tilde{X}}_{i\Delta_{n}})K_{h_{n}}(y-{\tilde{X}}_{(i+1)\Delta_{n}}),$$

$$B(x)=\sum_{i=1}^{n}{\omega_{i}}^{RNW}(x)K_{h_{n}}(x-{\tilde{X}}_{i\Delta_{n}}).$$

By Lemma 4, we have


$${\hat{p}}_{0}(y|x)-p(y|x)=\frac{1}{2}\sigma_{K}^{2}h_{n}^{2}\left(\frac{\partial^{2}p(y|x)}{\partial x^{2}}+\frac{\partial^{2}p(y|x)}{\partial y^{2}}\right)+O_{p}((\sqrt{nh_{n}^{2}}{)}^{-1}).$$

Thus, in order to prove Theorem 1(i)


$$\hat{p}(y|x)-p(y|x)=\frac{1}{2}\sigma_{K}^{2}h_{n}^{2}\left(\frac{\partial^{2}p(y|x)}{\partial x^{2}}+\frac{\partial^{2}p(y|x)}{\partial y^{2}}\right)+O_{p}((\sqrt{nh_{n}^{2}}{)}^{-1}),$$

we only need to prove


$$\hat{p}(y|x)-{\hat{p}}_{0}(y|x)=\frac{A(x,y)}{B(x)}-\frac{A_{0}(x,y)}{B_{0}(x)}\overset{P\;}{\to }0.$$

According to [8] and [22], we have $B_{0}(x)\overset{P}{\to }p(x)$, $B(x)\overset{P}{\to }B_{0}(x)$. In fact, according to Lemma 2, when $\sqrt{\Delta_{n}}/h_{n}\to 0$, the difference between ${\tilde{X}}_{i\Delta_{n}}$ and $X_{i\Delta_{n}}$ has a negligible impact on the kernel function, and by combining the asymptotic properties of the weight function (Lemma 3), it can be concluded that $\omega_{i}^{RNW}(x)\overset{P}{\to }\omega_{i}^{0}(x)$. Therefore, by Dominated Convergence Theorem, we can obtain $B(x)\overset{P}{\to }B_{0}(x)$.

So it is sufficient to prove that


$$A(x,y)\overset{P\;}{\to }A_{0}(x,y),\;(4)$$

For (4), we have


$$\begin{array}{c} \;\;\;A(x,y)-A_{0}(x,y)\\ =\sum_{i=1}^{n}\left[{\omega_{i}}^{RNW}(x)K_{h_{n}}(x-{\tilde{X}}_{i\Delta_{n}})-{\omega_{i}}^{0}(x)K_{h_{n}}(x-X_{i\Delta_{n}})]K_{h_{n}}(y-{\tilde{X}}_{(i+1)\Delta_{n}}\right)\\ \;\;\;\text{ + }\sum_{i=1}^{n}{\omega_{i}}^{0}(x)K_{h_{n}}(x-X_{i\Delta_{n}})\left[K_{h_{n}}(y-{\tilde{X}}_{(i+1)\Delta_{n}})-K_{h_{n}}(y-X_{(i+1)\Delta_{n}})\right]. \end{array}$$

Thus, we need to prove


$$\sum_{i=1}^{n}\left[{\omega_{i}}^{RNW}(x)K_{h_{n}}(x-{\tilde{X}}_{i\Delta_{n}})-{\omega_{i}}^{0}(x)K_{h_{n}}(x-X_{i\Delta_{n}})]K_{h_{n}}(y-{\tilde{X}}_{(i+1)\Delta_{n}}\right)\to 0,\;(5)$$

$$\sum_{i=1}^{n}{\omega_{i}}^{0}(x)K_{h_{n}}(x-X_{i\Delta_{n}})[K_{h_{n}}(y-{\tilde{X}}_{(i+1)\Delta_{n}})-K_{h_{n}}(y-X_{(i+1)\Delta_{n}})]\to 0.\;(6)$$

From [8], we have


$${\omega_{i}}^{RNW}(x)=\frac{1}{n(1+\lambda (x-{\tilde{X}}_{\left(i+1\right)\Delta_{n}})K_{h_{n}}(x-{\tilde{X}}_{i\Delta_{n}}))}$$

$$=\frac{1}{n}(1-\lambda (x-{\tilde{X}}_{\left(i+1\right)\Delta_{n}})K_{h_{n}}(x-{\tilde{X}}_{i\Delta_{n}})\;\;\;\;+\lambda^{2}((x-{\tilde{X}}_{\left(i+1\right)\Delta_{n}})K_{h_{n}}(x-{\tilde{X}}_{i\Delta_{n}}){)}^{2})+O(h_{n}^{3}),$$

and by Lemma 4,


$${\omega_{i}}^{0}(x)=\frac{1}{n(1+\lambda_{0}(x-X_{\left(i+1\right)\Delta_{n}})K_{h_{n}}(x-X_{i\Delta_{n}}))}$$

$$=\frac{1}{n}(1-\lambda_{0}(x-X_{i\Delta_{n}})K_{h_{n}}(x-X_{i\Delta_{n}})+{\lambda_{0}}^{2}((x-X_{i\Delta_{n}})K_{h_{n}}(x-X_{i\Delta_{n}}){)}^{2})+O(h_{n}^{3}).$$

(6) can be obtained through Lemma 2, and the details can be found in [3, p. 894]. For (5), it is equivalent to proving


$$\frac{1}{n}\sum_{i=1}^{n}\left[K_{h_{n}}(x-{\tilde{X}}_{i\Delta_{n}})-K_{h_{n}}(x-X_{i\Delta_{n}})\right]K_{h_{n}}(y-{\tilde{X}}_{(i+1)\Delta_{n}})\to 0,\;(7)$$

$$\begin{array}{c} \frac{1}{n}\sum_{i=1}^{n}K_{h_{n}}(y-{\tilde{X}}_{(i+1)\Delta_{n}})\lambda (x-{\tilde{X}}_{(i+1)\Delta_{n}}){\left(K_{h_{n}}(x-{\tilde{X}}_{i\Delta_{n}})\right)}^{2}\\ -\frac{1}{n}\sum_{i=1}^{n}K_{h_{n}}(y-{\tilde{X}}_{(i+1)\Delta_{n}})\lambda_{0}(x-X_{i\Delta_{n}}){\left(K_{h_{n}}(x-X_{i\Delta_{n}})\right)}^{2}\to 0, \end{array}\;(8)$$

$$\begin{array}{c} \frac{1}{n}\sum_{i=1}^{n}K_{h_{n}}(y-{\tilde{X}}_{(i+1)\Delta_{n}})\lambda^{2}{\left(x-{\tilde{X}}_{(i+1)\Delta_{n}}\right)}^{2}{\left(K_{h_{n}}(x-{\tilde{X}}_{i\Delta_{n}})\right)}^{3}\\ -\frac{1}{n}\sum_{i=1}^{n}K_{h_{n}}(y-{\tilde{X}}_{(i+1)\Delta_{n}}){\lambda_{0}}^{2}{\left(x-X_{i\Delta_{n}}\right)}^{2}{\left(K_{h_{n}}(x-X_{i\Delta_{n}})\right)}^{3}\to 0. \end{array}\;(9)$$

(7) can be obtained through Lemma 2, the proofs of (8) and (9) are similar, so the following only proves (8).


$$\frac{1}{n}\sum_{i=1}^{n}K_{h_{n}}(y-{\tilde{X}}_{(i+1)\Delta_{n}})(x-{\tilde{X}}_{(i+1)\Delta_{n}})\left[\lambda {\left(K_{h_{n}}(x-{\tilde{X}}_{i\Delta_{n}})\right)}^{2}-\lambda_{0}{\left(K_{h_{n}}(x-X_{i\Delta_{n}})\right)}^{2}\right]\to 0,\;(10)$$

$$\frac{1}{n}\sum_{i=1}^{n}K_{h_{n}}(y-{\tilde{X}}_{(i+1)\Delta_{n}})\lambda_{0}{\left(K_{h_{n}}(x-X_{i\Delta_{n}})\right)}^{2}\left[\left(x-{\tilde{X}}_{(i+1)\Delta_{n}})-(x-X_{i\Delta_{n}}\right)\right]\to 0.\;(11)$$

where (10) can be obtained through Lemma 2. For (11), by Lemma 1 and Lemma 3, we can get


$$\begin{array}{c} \;\;\;E\left[\frac{1}{n}\sum_{i=1}^{n}K_{h_{n}}(y-{\tilde{X}}_{(i+1)\Delta_{n}})(K_{h_{n}}(x-X_{i\Delta_{n}}){)}^{2}\lambda_{0}\left(\left(x-{\tilde{X}}_{(i+1)\Delta_{n}})-(x-X_{i\Delta_{n}}\right)\right)\right]\\ =O(h_{n})E\left[K_{h_{n}}(y-{\tilde{X}}_{(i+1)\Delta_{n}})(K_{h_{n}}(x-X_{i\Delta_{n}}){)}^{2}E\left[\left(\left(x-{\tilde{X}}_{(i+1)\Delta_{n}})-(x-X_{i\Delta_{n}}\right)\right)|X_{i\Delta_{n}}\right]\right]\\ =\frac{-\Delta_{n}O(h_{n})}{2}E\left[K_{h_{n}}(y-{\tilde{X}}_{(i+1)\Delta_{n}})(K_{h_{n}}(x-X_{i\Delta_{n}}){)}^{2}\mu (X_{i\Delta_{n}})\right]\text{ + }O(\Delta_{n})\\ =O(\Delta_{n}), \end{array}$$

where the second-to-last equation can be inferred as follows:


$$\begin{array}{c} \;\;\;E\left[\left(\left(x-{\tilde{X}}_{(i+1)\Delta_{n}})-(x-X_{i\Delta_{n}}\right)\right)|X_{i\Delta_{n}}\right]\\ =E\left[X_{i\Delta_{n}}|X_{i\Delta_{n}}\right]-E\left[\frac{Y_{\left(i+1\right)\Delta_{n}}-Y_{i\Delta_{n}}}{\Delta_{n}}|X_{i\Delta_{n}}\right]\\ =X_{i\Delta_{n}}-\frac{1}{\Delta_{n}}\left[X_{i\Delta_{n}}\Delta_{n}+\frac{1}{2}\mu \left(X_{i\Delta n}\right)\Delta_{n}^{2}\right]+O\left(\Delta_{n}\right)\\ =\frac{-\Delta_{n}}{2}\mu \left(X_{i\Delta n}\right)+O\left(\Delta_{n}\right). \end{array}\;(\text{ 12})$$

In conclusion,


$$\underset{n\to \infty }{\lim}E\left[\frac{1}{n}\sum_{i=1}^{n}K_{h_{n}}(y-{\tilde{X}}_{(i+1)\Delta_{n}})(K_{h_{n}}(x-X_{i\Delta_{n}}){)}^{2}\lambda_{0}\left(\left(x-{\tilde{X}}_{(i+1)\Delta_{n}})-(x-X_{i\Delta_{n}}\right)\right)\right]=0.$$

On the other hand,


$$\begin{array}{c} \;\;\;Var\left[\frac{1}{n}\sum_{i=1}^{n}K_{h_{n}}(y-{\tilde{X}}_{(i+1)\Delta_{n}}){\left(K_{h_{n}}(x-X_{i\Delta_{n}})\right)}^{2}(\lambda_{0}(x-{\tilde{X}}_{(i+1)\Delta_{n}})-\lambda_{0}(x-X_{i\Delta_{n}}))\right]\\ =\frac{\Delta_{n}O(h_{n})}{nh_{n}^{4}}Var\left[\sqrt{\frac{1}{n}}\sum_{i=1}^{n}h_{n}^{2}K_{h_{n}}(y-{\tilde{X}}_{(i+1)\Delta_{n}}){\left(K_{h_{n}}(x-X_{i\Delta_{n}})\right)}^{2}\frac{1}{\sqrt{\Delta_{n}}}(X_{i\Delta_{n}}-{\tilde{X}}_{(i+1)\Delta_{n}})\right]\\ =\frac{\Delta_{n}O(h_{n})}{nh_{n}^{4}}Var\left[\frac{1}{\sqrt{n}}\sum_{i=1}^{n}g_{i}\right], \end{array}$$

where $g_{i}=h_{n}^{2}K_{h_{n}}(y-{\tilde{X}}_{(i+1)\Delta_{n}}){\left(K_{h_{n}}(x-X_{i\Delta_{n}})\right)}^{2}\frac{1}{\sqrt{\Delta_{n}}}(X_{i\Delta_{n}}-{\tilde{X}}_{(i+1)\Delta_{n}})$. Then


$$Var\left[\frac{1}{\sqrt{n}}\sum_{i=1}^{n}g_{i}\right]\text{ = }\frac{1}{n}\sum_{i=1}^{n}Var(g_{i})+\frac{2}{n}\sum_{i=k+1}^{n}\sum_{k=1}^{n-1}Cov(g_{i},g_{k}),$$

with the same arguments as [3, p. 896], in order to prove $Var\left[\frac{1}{\sqrt{n}}\sum_{i=1}^{n}g_{i}\right]<\infty$, we only need to prove $E({g_{i}}^{2})<\infty$. In fact, by Lemma 1,


$$\begin{array}{c} \;\;\;\frac{1}{\Delta_{n}}E\left[{\left({\tilde{X}}_{(i+1)\Delta_{n}}-X_{i\Delta_{n}}\right)}^{2}|X_{i\Delta_{n}}\right]\\ =\frac{1}{\Delta_{n}}\left[E\left({\tilde{X}}_{(i+1)\Delta_{n}}^{2}|X_{i\Delta_{n}}\right)+E\left(X_{i\Delta_{n}}^{2}|X_{i\Delta_{n}}\right)-2E\left({\tilde{X}}_{(i+1)\Delta_{n}}X_{i\Delta_{n}}|X_{i\Delta_{n}}\right)\right]\\ =\frac{1}{\Delta_{n}}\left[E\left(\frac{{\left(Y_{(i+1)\Delta_{n}}-Y_{i\Delta_{n}}\right)}^{2}}{\Delta_{n}^{2}}|X_{i\Delta_{n}}\right)+E\left(X_{i\Delta_{n}}^{2}|X_{i\Delta_{n}}\right)-2E\left(\frac{Y_{\left(i+1\right)\Delta_{n}}-Y_{i\Delta_{n}}}{\Delta_{n}}X_{i\Delta_{n}}|X_{i\Delta_{n}}\right)\right]\\ =\frac{1}{\Delta_{n}}[X_{i\Delta_{n}}^{2}+\left(\frac{1}{3}\sigma^{2}\left(X_{i\Delta_{n}}\right)+X_{i\Delta_{n}}\mu \left(X_{i\Delta_{n}}\right)\right)\Delta_{n}+X_{i\Delta_{n}}^{2}\\ \;\;\;-2(X_{i\Delta_{n}}^{2}+\frac{1}{2}\mu \left(X_{i\Delta n}\right)\Delta_{n}X_{i\Delta_{n}})+O(\Delta_{n}^{2})]\\ =\frac{1}{3}\sigma^{2}\left(X_{i\Delta_{n}}\right)+O(\Delta_{n}), \end{array}\;(13)$$

so we can get $E({g_{i}}^{2})<\infty$. Since Δn/Δn(nhn4)(nhn4)→0, we have


$$\underset{n\to \infty }{\lim}Var\left[\frac{1}{n}\sum_{i=1}^{n}K_{h_{n}}(y-{\tilde{X}}_{(i+1)\Delta_{n}}){\left(K_{h_{n}}(x-X_{i\Delta_{n}})\right)}^{2}\lambda_{0}\left(\left(x-{\tilde{X}}_{(i+1)\Delta_{n}})-(x-X_{i\Delta_{n}}\right)\right)\right]=0.$$

(ii) Next, we will prove the asymptotic normality of the new estimator. Let


$$A_{n}=\sqrt{nh_{n}^{2}}\left({\hat{p}}_{0}(y|x)-p(y|x)-\frac{1}{2}\sigma_{K}^{2}h_{n}^{2}\left(\frac{\partial^{2}p(y|x)}{\partial x^{2}}+\frac{\partial^{2}p(y|x)}{\partial y^{2}}\right)\right),$$

$$B_{n}=\sqrt{nh_{n}^{2}}(\hat{p}(y|x)-{\hat{p}}_{0}(y|x)).$$

By Lemma 4, we have


$$A_{n}\overset{D}{\to }N\left(0,\frac{R^{2}(K)p(y|x)}{p(x)}\right).$$

From Theorem 1(i), the approximation error satisfies:


$$\hat{p}(y|x)-{\hat{p}}_{0}(y|x)=O_{p}(\Delta_{n}),$$

and under the condition of Theorem 1(ii) ($nh_{n}^{2}\Delta_{n}^{2}\to 0$), we have


$$B_{n}=\sqrt{nh_{n}^{2}}\cdot O_{p}(\Delta_{n})=O_{p}(\sqrt{nh_{n}^{2}}\Delta_{n})=o_{p}(1).$$

Therefore, by Slutsky’s theorem, the sum converges


$$A_{n}+B_{n}\overset{D}{\to }N\left(0,\frac{R^{2}(K)p(y|x)}{p(x)}\right).$$

This completes the proof.

## Conclusion

This study considers nonparametric estimation for second-order diffusion processes by developing a novel re-weighted estimator of transition probability density function based on the Nadaraya-Watson kernel estimation and local linear estimation methods. The core of the re-weighted idea is to redistribute weights to follow the prerequisite of local linear estimation without changing the inherent properties of the Nadaraya-Watson estimator. The proposed estimator not only preserves the nonnegativity of density estimation but also incorporates the bias advantage of local linear estimation. In addition, the consistency and asymptotic properties of the estimator under mild conditions are established.

In defining the re-weighted estimator of transition probability density, we used the same bandwidth *h*_*n*_ for both the *x*-direction and *y*-direction, the purpose of using a single bandwidth is to simplify the presentation of asymptotic properties (e.g., bias, variance, and asymptotic normality) of the re-weighted estimator, this is consistent with the simplification strategy used in [14] to focus on the essential innovation of the re-weighted method. In the simulation, the state variable ${\tilde{X}}_{i\Delta_{n}}$ of the second-order diffusion process is approximated by the difference of observation $Y_{i\Delta_{n}}$, if there are differences in the variability of ${\tilde{X}}_{i\Delta_{n}}$ across different observation intervals (e.g., $\Delta_{n}=0.002$ and $\Delta_{n}=0.0005$), ${\tilde{X}}_{i\Delta_{n}}$ can be standardized first (with mean 0 and variance 1), and then the single bandwidth $h_{n}=1.06Sn^{-1/6}$ (where *S* is the sample standard deviation after standardization) can be used to ensure the smoothing scale adaptation to the joint distribution characteristics of the two variables. In practical applications, however, it may be necessary to apply different levels of smoothing to each direction. For example, in paleotemperature sequences, if the variability difference between *x* (current paleotemperature value) and *y* (subsequent paleotemperature value) is significant, resulting in the need for differentiated smoothing even after standardization. Under such circumstances, the re-weighted estimator in this paper can be redefined into a dual-bandwidth form: introducing the bandwidth *h*_2,*n*_ for the *x*-direction (corresponding to $K_{h_{2,n}}(x$ − ${\tilde{X}}_{i\Delta_{n}})$) and the bandwidth *h*_1,*n*_ for the *y*-direction (corresponding to $K_{h_{1,n}}(y$ − ${\tilde{X}}_{(i+1)\Delta_{n}})$) respectively in the weight calculation. At this time, the nonnegativity and low-bias characteristics of the estimator can still be retained through the original re-weighted mechanism, and only the convergence order conditions of the bandwidth in the asymptotic analysis need to be adjusted (e.g., changing $nh_{n}^{2}\to \infty$ to $nh_{1,n}h_{2,n}\to \infty$).

To enhance the practical applicability of the proposed method in the real world, delving into practical considerations like computational complexity, parameter sensitivity analysis, and performance under more complicated real world data scenarios (e.g., noisy data, irregular sampling intervals) using systematic empirical methods is crucial. This would involve investigating how our method performs compared with existing approaches in handling these practical challenges, and this paper does not conduct such in-depth practical investigations. However, comprehensively tackling these real-world application problems remains a promising direction for future research, being able to more robustly validate the practicality of our method in real-world operating environments.

Theoretical analysis and simulation studies jointly verify the superiority of this estimator. Theoretical analysis shows that the bias of the proposed estimator is indeed smaller than that of the Nadaraya-Watson estimator. And simulation experiments validated these findings, further confirming the advantages of our method.
